# Retraction: MicroRNA-34a regulates liver regeneration and the development of liver cancer in rats by targeting Notch signaling pathway

**DOI:** 10.18632/oncotarget.28676

**Published:** 2024-11-22

**Authors:** Xiao-Ping Wang, Jian Zhou, Ming Han, Chuan-Bao Chen, Yi-Tao Zheng, Xiao-Shun He, Xiao-Peng Yuan

**Affiliations:** ^1^Third Division of Organ Transplant Center, The Eastern Hospital of The First Affiliated Hospital, Sun Yat-sen University, Guangzhou 510700, P. R. China; ^*^These authors contributed equally to this work

**This article has been retracted:** Corresponding author Xiao-Peng Yuan contacted Oncotarget and stated there are ‘major flaws’ in the paper. Oncotarget requested that the Academic Committee at Sun Yat-sen University review this paper. Finally, the Supervision Committee of the National Natural Science Foundation of China reviewed the paper. This review discovered problems, such as writing by a third-party company for investment, false signature, and unauthorized marking of the National Natural Science Foundation of China project number. The corresponding author Yuan Xiaopeng is responsible for the above problems. In addition, Yuan Xiaopeng included this paper in his application for the National Natural Science Foundation of China (application number 8177030286), and he should also be responsible for the objective results of false information in the application. In addition, Figure 11B contains numerous image duplications with Figure 12B of [1], which has since been retracted. Except the corresponding author, all other authors were unable to be reached. Therefore, the Editorial decision was made to retract the paper.

Original article: Oncotarget. 2017; 8:13264–13276. 13264-13276. https://doi.org/10.18632/oncotarget.14807
